# Mechanisms of impact of alcohol availability interventions from the perspective of 63 diverse alcohol licensing stakeholders: a qualitative interview study

**DOI:** 10.1080/09687637.2023.2205991

**Published:** 2023-05-04

**Authors:** R. O’Donnell, A. Mohan, R. Purves, N. Maani, C. Angus, M. Egan, N. Fitzgerald

**Affiliations:** a Institute for Social Marketing and Health, Faculty of Health Sciences and Sport, University of Stirling, Stirling, UK; b School of Health Sciences, University of Dundee, Dundee, UK; c School of Social and Political Science, The University of Edinburgh, Edinburgh, UK; d School of Health and Related Research, University of Sheffield, Sheffield, UK; e Department of Public Health, Environments and Society, London School of Hygiene and Tropical Medicine, London, UK; f SPECTRUM Consortium, Edinburgh, UK

**Keywords:** Alcohol availability, alcohol premises licensing, public health, mechanisms of change, outlet density, opening hours

## Abstract

**Aims:**

Interventions restricting temporal and spatial availability of alcohol are associated with reduced harm, but the pathways by which specific interventions have impact are poorly understood. We examined mechanisms of impact from the perspective of diverse licensing stakeholders.

**Methods:**

Fifty-three in-depth interviews were conducted with licensing stakeholders (from public health teams [PHTs], police, local authority licensing teams and lawyers, and alcohol premises licensing committees) from 20 local government areas. Interviewees were recruited as part of the Exploring the impact of alcohol licensing in England and Scotland (ExILEnS) study. Data were analyzed thematically and preliminary themes/subthemes were discussed during online groups with a different sample of public health and licensing professionals (*n* = 10).

**Findings:**

Most interviewees struggled to articulate how availability interventions might lead to changes in alcohol consumption or harms. Five overarching mechanisms were identified: access, visibility, premises and area-level norms, affordability, and management of the night-time economy, with specific pathways identified for certain subgroups/premises types. The mechanisms by which alcohol availability interventions may impact on alcohol consumption and harms are diverse, but were poorly understood.

**Conclusions:**

These findings will inform licensing and availability policy and advocacy, highlighting the need for further scrutiny of the evidence underpinning identified mechanisms, and primary research to address knowledge gaps.

## Introduction

Multiple systematic reviews find that measures reducing the physical and temporal availability of alcohol are associated with lower alcohol-related harms (Babor et al., 2022; Bryden et al., 2012; Campbell et al., 2009; Middleton et al., 2010; Public Health England, 2016; Sherk et al., 2018). Such reviews draw on studies in diverse jurisdictions worldwide, suggesting that the broad principle that more availability will tend to give rise to greater alcohol consumption and harms is likely to be sound. The World Health Organization strongly recommends reducing availability as a means of addressing alcohol harms, and whilst there are several other effective strategies that can also impact, including taxation and other pricing strategies (e.g. minimum unit pricing [MUP]–introduced in Scotland in 2018) and restrictions on marketing (Babor et al., 2022), most are not under local control (Fitzgerald et al., 2017). Reducing availability is not straightforward in practice, especially in relatively permissive licensing regimes in the UK and elsewhere, and mechanisms of effect are not well understood (Gmel et al., 2016; Holmes et al., 2014).

Under slightly different systems in England and Scotland, local licensing committees of elected local politicians consider applications for licences to sell alcohol (Fitzgerald et al., 2023b) and must produce a ‘Statement of Licensing Policy’ (SLP) every five years outlining priorities and how they will achieve the statutory licensing objectives. These objectives include preventing crime, disorder and public nuisance, promoting public safety, protecting children from harm, and in Scotland only, protecting and improving public health (Scottish Parliament, 2005; UK Parliament, 2003).

SLPs must include a statement on ‘overprovision’; that is, whether there are areas with an excessive number of outlets, or of a particular type of outlet, based on evidence. In England, licensing authorities can designate areas as ‘Cumulative Impact Zones’ (CIZs), where evidence indicates that the number, type or density of licensed premises impacts adversely on the licensing objectives. In overprovision or CIZ areas, licence applications can be rejected unless applicants demonstrate why their application should be granted. In both nations, it is theoretically possible to cap, but not reduce the number of licensed premises selling alcohol in an area. SLPs can also set preferred operating hours. In England, it is possible for on- and off-licence premises to apply to sell alcohol 24 h a day. In Scotland, alcohol is sold for off-premises consumption between the hours of 10 am and 10 pm only. Licensing Boards must be satisfied that exceptional circumstances apply to grant a 24 h licence for an on-sales premise.

Under the legislation in both nations, stakeholders including public health and the police have a statutory role in the licensing system. They are informed of new applications and can respond by making an objection or representation designed to lead to amendments to, or the refusal of, an application. They are also consulted on local licensing policy. All such stakeholders therefore need to understand and articulate how licensing policies and decisions may impact on the licensing objectives. They are well-placed to have insight into the pathways by which local changes in spatial and temporal availability might impact on alcohol consumption, intoxication, health, crime, disorder, etc. Understanding these mechanisms would likely be beneficial to enable development of a compelling case to local licensing authorities. Clear, convincing stories (‘narratives’) for policymakers are considered an effective strategy used by advocates seeking policy change, in theory (McBeth et al., 2014), and in practice, e.g. in relation to MUP for alcohol (Katikireddi et al., 2014). Research suggests some local policymakers are less influenced by academic evidence in relation to local alcohol licensing, are skeptical about the transferability of international evidence, and show a preference instead for anecdote and local experience to inform local policy and decision making (Fitzgerald et al., 2017; Fitzgerald & Cairney, 2022; Reynolds et al., 2019).

To our knowledge, no published research has directly explored licensing stakeholders’ views on the mechanisms by which alcohol availability interventions may impact on alcohol harms. Given the potential importance of narratives in explaining the rationale for local licensing policies and decisions, and gaps in current literature articulating local mechanisms of change for availability interventions, our aim was to explore licensing stakeholders’ views on the potential pathways by which temporal and physical availability interventions may lead to impacts on alcohol-related health harms, crime, disorder, and other outcomes. This exploration involved consideration of what mechanisms and pathways to change stakeholders assumed to operate, whether the stakeholders felt confident that those pathways really did lead to impacts, and what stakeholders appeared to base their opinions on.

## Materials and methods

Data collection included in-depth interviews (*n* = 53) and online discussion groups (*n* = 10).

### Context, overview, and ethics

We present findings from in-depth interviews and online discussion groups as part of the ‘Exploring the impact of licensing in England and Scotland (ExILEnS)’ study (Fitzgerald et al., 2018a). ExILEnS was approved by the University of Stirling Ethics Committee for NHS, Invasive or Clinical Research (NICR 16/17–64 and 064 A) and the London School of Hygiene and Tropical Medicine Observational/Interventions Research Ethics Committee (14283). NHS Research and Development approval was secured from all participating NHS Boards in Scotland. This was not required for public health teams (PHTs) in England based within local government. Full informed consent was obtained from all participants, who were assured of confidentiality. Interviews and discussions were audio-recorded and transcribed verbatim.

### Recruitment, data collection, and analysis

#### In-depth interviews

In accordance with the ExILEnS protocol (Fitzgerald et al., 2018a), we recruited 20 PHTs who had been actively seeking to engage in alcohol premises licensing: 14 in England; 6 in Scotland. Within these 20 areas, potential interviewees (PHT members, licensing officers/managers, police staff with a licensing remit, elected members, and licensing lawyers/clerks) were identified through direct contact, snowball sampling, and site visits. Fifty-three telephone interviews were conducted (by RO, NM, AM, and RP) between November 2018 and October 2020, with 28 PHT members, nine licensing officers/managers, six police staff with a licensing remit, five elected members and five licensing lawyers.

Separate topic guides were developed for each of the five stakeholder groups, as this was part of a larger study which also explored interviewee roles, responsibilities and purpose in the licensing system (findings have been published separately, alongside a detailed description of the role and remit of key stakeholder groups in the licensing system, for which there are differences in Scotland and England (Fitzgerald et al., 2023b; O’Donnell et al., 2022)) Each topic guide was based on alcohol licensing literature and team discussion, including open-ended questions (Supplementary Table 1) to elicit views on the ways in which temporal and spatial availability interventions impact on a range of alcohol-related harms. In total, 29 interviewees were based in England and 24 were based in Scotland.

Interviews lasted between 32 min and 2 h 36 min (median = 1 h 12 min). Using NVivo 12, RO, AM, and RP coded anonymized transcripts against a set of categories created using deductive (reviewing research questions) and inductive approaches (reading transcripts). Each category was developed iteratively, with ongoing refinements made while re-examining the data and based on team discussions. Relevant extracts were then reviewed by RO and NF. RO wrote up interim analysis findings, which were refined in discussion with NF, ME, AM, and RP.

#### Online discussion groups

RO, NF, and ME conducted two small discussion groups (*n* = 10) with additional stakeholders with expertise in public health and licensing identified through the study team and steering committee, and via snowball sampling. Groups were asked how a range of temporal and spatial availability interventions might impact on alcohol-related harms, and possible mechanisms of change involved, including preliminary findings from in-depth interviews (Supplementary Table 2 provides a summary of questions). The groups lasted 1 h 40 and 1 h 51 min. RO read and re-read both transcripts to identify extracts providing confirmatory and contradictory insights compared to the in-depth interview findings, which were discussed with NF and ME. These extracts informed further analysis of in-depth interview data.

## Results

We first report on the ease with which participants responded to questions about mechanisms of impact of temporal and spatial availability, and then outline five identified categories of (potential) mechanism of positive or negative impact of changes in alcohol availability (see Table 1). For each category, we outline with illustrative quotes individual potential mechanisms discussed, population subgroups for whom the mechanism is thought to apply (where relevant), and any suggested factors outlined that could moderate this mechanism. The mechanisms discussed are all described as mediated through an overt or implied impact on alcohol consumption.

**Table 1.** Identified categories of mechanism of change for temporal and spatial availability interventions aimed at reducing alcohol-related harms.

Identified categories of mechanisms of change
*1. Access:* Shaping consumption through ease/convenience of access or removal of access to alcohol
2. *Visibility:* Shaping consumption and/or consumption norms through visibility of alcohol (including pathways via drinking cues and normalization of drinking)
3. *Premises and area-level norms:* Shaping norms of consumption or behavior at premises or area level, through premises type and operation
4. *Affordability*: shaping consumption through pricing including availability of high strength/low cost alcohol
5. *Management of the night-time economy*: shaping harms through manipulating the late night environment (via staggered closing times)

Participants were largely unused to thinking systematically about explanatory mechanisms for availability interventions. Almost all licensing and public health stakeholders found it challenging to articulate the ways in which temporal and spatial availability interventions might impact on alcohol-related harms, needing time to consider, and formulate their views. There were no apparent differences between public health and other licensing stakeholders outside of health in terms of ability to articulate potential mechanisms, or mechanisms discussed. When stakeholders explained their views on how availability interventions impact on harms, they often referred to the existing evidence base on spatial availability:

“That’s a difficult one for me to answer. Certainly there are several health studies which link the number and capacity of premises in an area to the harms that are experienced within it. I don’t think I could answer that much further than that, to be honest.” (Licensing Standards Officer, Scotland)

Stakeholders were often less confident postulating how proposed mechanisms of change might apply to their own locality and to different population subgroups:

“These are quite big [questions about mechanisms of change] and being a Public Health professional I’m finding myself wringing my hands thinking I’m not sure I have any evidence to support this [suggestion]!” [laughs] (Assistant Director of Public Health, England)

The strongest views, and those that participants felt more confident expressing, related to a perceived causal link between *increased* availability and increased alcohol consumption. Several interviewees focused on the impacts associated with *extending* opening hours in the night-time economy, citing well-established links between extended hours, increased alcohol consumption, and increased strain on emergency services (see below). Some talked of perceived impact, e.g. on emergency services, without actually articulating a mechanism by which that occurred.

Interviewees were generally less comfortable identifying mechanisms by which reduced availability would impact positively on alcohol-related harms. Several felt there was no association between reduced opening hours and reduced harms, and whilst most made no reference to existing evidence on temporal availability, a few public health stakeholders wrongly perceived the international evidence shows "*minimal to no evidence"* of an association (Public Health Specialist, Public Health Officer, both England).

Both discussion groups suggested that local geographical context including variations in service provision, rurality/urbanity and area level deprivation would be likely to affect the pathways by which spatial and temporal availability interventions might impact on alcohol-related harms. Such pathways were felt to be complex and could include ‘ripple’ effects, such as adaptations to consumer behavior or effects in neighboring areas.

(1) Access: shaping consumption through ease/convenience of access or removal of access to alcohol

This was the most straightforward mechanism discussed by participants, who linked greater access to alcohol to greater consumption and harms. First, some public health stakeholders spoke of the potential for increased spatial availability to lead to increased alcohol consumption as a result of the ‘*convenience factor*’, e.g. when discussing the proximity of off-licence stores to individuals and the potential influence this has on consumer behavior. Greater proximity was felt to remove/reduce one barrier to purchase–the distance to travel:

"*If there was an off-licence next door, I would possibly pop in there far more than I do now because it’s a walk to go there*." (Substance Misuse Team, England)

Second, participants discussed how restrictions on temporal availability put controls on people’s access to alcohol in situations where people were perceived as unable to control their own drinking. In particular, a few public health stakeholders noted the importance of reduced accessibility to alcohol, related to restricting off-licence opening hours in the morning, as one possible means of reducing early morning alcohol consumption for street drinkers and people with alcohol dependence:

“So the amount of time that you’re able to buy alcohol will determine how many hours you’re drinking it…If they [people with alcohol dependence] can drink it from 7 o’clock in the morning they will. And if they can’t…its imposing control on peoples’ drinking, people who haven’t got any control on it, so I think on that level at least the opening hours is significant.” (Senior Public Health Manager, England)

Similarly, a few interviewees felt that capping availability in locations near alcohol treatment services would reduce accessibility (and visibility, see below) of alcohol for people with alcohol dependence:

“The cumulative impact zone, was included in the area of our treatment centres for people suffering with alcohol misuse…it was about respecting that there was an alcohol misuse problem in this community that actually by reducing [meaning preventing any increase in] the availability of alcohol would allow us to start really making inroads…” (Head of Public Health Programmes, England)

Third, one participant spoke about later opening hours encouraging people to stay out later than they otherwise would, thus leading to them drinking more alcohol. There is an inevitability implied in the description here rather than an explicit mechanism–a sense that if people can drink later, they will–though the participant does not explicitly discuss this in terms of loss of control over drinking.

“So undoubtedly extended licensing hours, not intentionally, but it does promote the increased consumption of alcohol because people will go out and their end goal is to get to that late night premises.” (Licensing Standards Officer, Scotland)

Whilst these mechanisms were clearly described for *increased* availability, there was a sense that attempts to reduce harm through *restrictions* on availability may not be successful due to several potential moderators of this mechanism category. Most importantly, participants felt that drinkers would find alternative temporal or spatial sources of alcohol. In England, several interviewees suggested that the potential for positive impacts of temporal restrictions on on-trade availability would be moderated/eliminated by the existence of 24-h off-licences as participants would be able to purchase alcohol from shops even if bars/clubs were shut.

“I’m not sure we’re particularly focused on opening times or particularly worried about 24 hour access to alcohol because it’s there anyway really. I think that boat has gone [ship has sailed].” (Health Protection team member, England)

One in-depth interviewee asserted that there could be unintended consequences of restricting opening hours in the evening, because "people panic drink", consuming the same quantity of alcohol in an evening, but over a shorter time-span (Licensing Sergeant, Scotland). Some interviewees suggested that any positive impacts of restricting spatial availability would be moderated by consumer adaptations. Some might walk longer distances or travel by car to purchase alcohol elsewhere, therefore "*the majority of sales would still occur, they would just take place within the number of premises that are open."*(Police Licensing Sergeant, Scotland).

Several interviewees suggested that people may instead access alcohol through online/app-based retailers or via home delivery services. One interviewee felt that "*the [online] sale of alcohol and delivery to home addresses…it’s actually alarming how big a business it’s becoming…it’s just making alcohol more accessible and that’s the worrying thing I think."*(Licensing Standards Officer, Scotland). Another suggested "*we need to look again at [the notion of] overprovision"* on this basis. (Health Improvement Officer, Scotland).

(2) Visibility: shaping consumption and/or consumption norms through visibility of alcohol (including pathways *via* drinking cues and normalization of drinking)

Participants described two potential mechanisms of impact arising from greater visibility of alcohol. First, increased spatial availability, whereby more premises would be seen by consumers, was suggested to increase consumption because each sighting could act as a reminder or ‘cue’ to drink alcohol. One interviewee described how visibility might combine with ease of access to increase purchasing (and therefore, it is implied, consumption) as it is "*almost a reminder for people…and it makes it so easy to purchase rather than something that perhaps you would buy with a weekly shop or at the weekend."* (Health Improvement Lead, Scotland).

The role of additional premises as ‘cues’ for drinking was also a concern given the challenge such cues pose in particular for individuals seeking to establish/maintain recovery from alcohol dependence. As noted above, this was sometimes cited as a reason to limit spatial availability (particularly of off-licence premises) in areas where treatment centers were sited.

The second potential mechanism relating to visibility discussed by participants was based on the idea that greater visibility of alcohol would lead to children’s normalization of alcohol consumption. A few interviewees discussed this in relation to restricting off-licence opening hours in the morning, which was felt to have a potentially important role in limiting children’s exposure to alcohol marketing and consumption during the morning school run:

“If you’ve got…off-licences opening very early in the morning, we’re trying to avoid children walking past them going to schools, we’re trying to protect the children.” (Licensing Lawyer, England)

The pathway to greater alcohol consumption and harm was not explicitly described here but would presumably play out over a longer time period than the other mechanisms described. Some participants in the online discussion group noted that they hadn’t previously considered de-normalization as a mechanism for impact of availability interventions, but welcomed hearing about it when presented with preliminary interview findings.

(3) Premises and area-level norms: Shaping norms of consumption or behavior at premises or area-level, through premises type and operation

Several public health stakeholders in England felt that certain types of premises were more or less likely to be associated with alcohol-related harms, because they enabled or promoted certain ‘norms’ of alcohol consumption and/or behavior. A few suggested that certain ‘vertical’ drinking establishments (with more people standing rather than sitting) encouraged heavy episodic drinking, Others suggested that louder dance-focused premises may be associated with greater disorder and violence. Reducing the numbers of such premises could therefore have a positive impact, whereas food-focused premises were felt to be less likely to increased consumption or disorder.

“If you are sitting down eating, sipping your drink slowly, you’re having conversations with people, you’re not going to do yourself the same alcohol-related impact as if you are standing up downing eight pints quite fast without food.’” (Alcohol Strategy Lead, England)

Participants also noted the potential to impact on *area-level norms* or ‘culture’ by preventing vertical drinking establishments from opening, but facilitating the opening of new food-based businesses. Such ‘place-shaping’ was also focused on off-licence premises, which were felt by some participants to be more problematic than on-licence premises, in part because off-licences tend to sell cheaper, high strength alcohol (see next section below) but also because on-trade venues offer a more controlled drinking environment–"the fact that alcohol is sold in measures, the fact that alcohol is generally a higher price……the police have spent a lot of time talking [with on-trade staff] about [customer] vulnerability and about duty of care. So there’s a lot of things that reduce the risk [of alcohol-related harms] with the on trade." *(*Consultant in Public Health, Scotland).

‘Place-shaping’ was felt to be something that could be done through CiZs and overprovision policies. Attempts to cap the number of off-licence premises, whilst promoting new restaurants in a local area were also viewed as aligned with economic development and area regeneration.

“The Cumulative Impact Zone is in place to try and prevent more off-licences from opening… …It’s about looking at whether [new outlets] are going to be responsible, how they’re going to impact? Are they going to add more quality…so for example there’s an out of town area with lots of restaurants that’ve opened, so that’s increased the number of places selling alcohol, but they’ve encouraged that more because that’s like more of a business and commercial opportunity and regeneration scheme…balancing business interests with the impact on the population.” (Alcohol Strategy Lead, England)

In the discussion groups, one participant questioned whether "*place shaping could be seen as discriminatory if the preference is for venues more typically associated with middle class consumers."*

(4) Affordability: shaping consumption through pricing including affordability of high strength/low cost alcohol

Several public health and licensing professional interviewees associated off-licence premises with greater alcohol consumption and harms than on-trade premises due to the greater affordability of alcohol available in the off-trade in general ("*There’s a lot of cheap alcohol in off-licences."* (Director of Public Health, England)).

The potential impact of temporal restrictions on off-licence sales (such as the limited opening hours–10am–10pm for off-licences in Scotland) was not discussed, although 24 h off-licence opening hours were thought to limit potential benefits from limiting on-trade sales in England (see above).

Interviewees in England felt that the availability of high strength, low cost alcohol contributes to greater consumption and harms including public health and crime and disorder especially amongst dependent/homeless drinkers. This category of products is not available in Scotland due to MUP.

“People can be buying alcohol from off-licences and…we can see the worst effects of it, particularly with the homeless side, because they are going in there to buy strong alcohol, which is at [a] very cheap price…and the pricing of sales in on-sale premises doesn’t lend itself to those same harms as it does in off-licences.” (Licensing Standards Officer, England)

(5) Management of the night-time economy: shaping harms through manipulating the late night environment (via staggered closing times)

The late-night availability of alcohol can also be manipulated through having staggered closing times for late-night on-trade premises rather than most premises closing at a fixed time. Some licensing stakeholders felt that staggered closing reduced the likelihood of public nuisance/disorder, by limiting the flow of customers exiting premises at the same time. One public health professional spoke of mixed views within their local licensing team on whether staggered closing times reduce strain on police services or extend the need for it:

“If there’s [more] people going home over a period of time as opposed to literally everybody is kicked out at 5 in the morning, …that is very tricky to manage although…others were saying that at least you knew where to focus your policing around those places that were kicking out at that time.” (Assistant Director of Public Health, England)

Others suggested staggered closing hours simply change the location of harms, because "*people will leave the one premises that closes at half 11 and then they’ll all march down the street to the one that closes at half 12."’* (Elected member, England)

## Discussion

Licensing and public health stakeholders found it challenging to spontaneously articulate with confidence specific pathways by which decreases in alcohol availability may impact on consumption and harms. They were largely unfamiliar with this mechanistic way of thinking. Some had a surface familiarity with available international evidence, discussing the existence of evidence linking outlet numbers to harm. However, none cited studies of the link between later opening hours and crime, despite this also being a strong feature of the international literature (Kypri et al., 2016; Rossow & Norström, 2012; Sanchez-Ramirez & Voaklander, 2018; Wilkinson et al., 2016). Stakeholders identified five overarching mechanisms: access, visibility, premises and area-level norms, affordability, and management of the night-time economy. Most of these encompassed more specific sub-mechanisms, or pathways specific to certain population subgroups including people with alcohol dependence and children. Whilst we could identify no previous studies with comparable data, the mechanisms suggested appear plausible, and are more diverse than typically studied or theorized for availability interventions, encompassing rationale more common to marketing/pricing interventions including drinking cues, normalization and affordability. We firstly discuss suggested mechanisms in relation to existing research evidence and theoretical literature, and then we consider implications for research, policy, and practice.

### Suggested mechanisms in relation to existing research evidence and theory

Participants were most comfortable discussing how *greater* ‘access/convenience’ arising from increased spatial or temporal availability could lead to greater alcohol consumption. They were generally less comfortable identifying mechanisms by which *reduced* availability would impact positively on alcohol-related harms. This may in part reflect time delays in any impact from temporal availability, in a context where spatial and online sales availability stay the same (as they cannot easily be reduced by the licensing system in the UK). This was recognized by our participants and highlights the need to consider how licensing systems address home delivery, app-based and online availability, especially following the COVID-19 pandemic (Fitzgerald et al., 2022; Reynolds & Wilkinson, 2020).

Some interviewees perceived that access controls were necessary to protect individuals with alcohol dependence, who were seen as having limited capacity to control their own drinking, however; they made little mention of the role intoxication might play in limiting the ability of late-night drinkers to do the same. The nature of alcohol as a drug that impairs decision-making and drives impulsivity (Adams et al., 2013; Lawrence et al., 2009) is critical to understanding why later opening hours are perceived by our interviewees to almost inevitably lead to continued drinking by people already under the influence, through purchases in late night on-trade premises, or from late night off-trade premises if on-trade premises closed earlier. Whilst it seems unlikely that all customers of late night bars/clubs would buy alcohol from off-sales premises if on-sales premises closed earlier, this illustrates the challenge PHTs face in seeking to reduce harms arising from availability in highly permissive licensing systems.

Considering visibility of alcohol, people in recovery from alcohol dependence in Scotland have highlighted the challenge to their recovery posed by ‘triggers’ from seeing alcohol outlets in their community and by 24 h availability (Shortt et al., 2017). Cue reactivity is the principle that mere exposure to cues associated with alcohol use (e.g. an alcohol outlet or seeing others drinking) can trigger physiological and psychological responses (Adams, 2013). Importantly, these responses are often automatic, occurring without any conscious thought, and can trigger alcohol seeking (Herrmann et al., 2001) and consumption (Greeley et al., 1993), especially in heavier, more regular drinkers (Cox et al., 1999). Regarding visibility and normalization of alcohol for children, in Scotland, the Children’s Parliament has reported that children do not want to be exposed to alcohol in or outside shops, and on the way to/from school (Alcohol Focus Scotland and the Children’s Parliament, 2019) and research from New Zealand suggests alcohol outlets may be an important source of exposure of children to alcohol marketing (Chambers et al., 2018).

Interviewees reported several mechanisms by which premises type might influence harms, including through premises- and area-level norms giving rise to greater or lesser alcohol consumption and harms in vertical drinking establishments and restaurants, respectively. These plausible pathways are inadequately examined in available evidence (Gmel et al., 2016; Holmes et al., 2014), in part because many studies aggregate different outlet types to larger categories (i.e. on-premises, off-premises, or even total outlets) (Gmel et al., 2016). Studies that have disaggregated on-trade outlets into clubs, bars, and pubs have found that fighting and sexual assault are associated with bars and nightclubs (Cameron et al., 2016; Gmel et al., 2016), perhaps because these premises are often located in high densities in night-time economy spaces (Horsefield et al., 2023). Some premises may be more likely to attract individuals who are at risk of committing violence, as per Gruenewald’s ‘niche theory.’ This theory posits that premises deliberately market themselves to specific social groups, leading to concentrations of drinkers in establishments with people like themselves, which in turn leads to elevated levels of harms in some outlet types, for example where heavy drinkers congregate (Gruenewald, 2007). Current theoretical literature relating to alcohol availability is largely limited in focus to explaining why specific outlets or clusters of outlets are associated with increased harm, especially crimes (i.e. routine activities theory (Cohen & Felson, 1979); niche theory (Gruenewald, 2007), social disorganization theory (Sampson et al., 1997; Skogan, 1990). Systematic scoping reviews to assess and critique available evidence supporting or undermining the mechanisms suggested would enable more comprehensive theories to be developed.

Considering affordability, in the absence of MUP in England, stakeholders discussed the potential impact of limiting the availability of cheap alcohol by limiting off-licence numbers, to address concerns about street drinking, and vulnerable dependent drinkers. MUP would address the availability of cheap alcohol much more effectively by raising the price of all such products (Stead et al., 2020). Recent attempts to address cheap alcohol through licensing have shown little or no benefit as they were limited to specific products, shops and areas, relied on retailers’ cooperation and led to displacement effects (McGill et al., 2016; Pliakas et al., 2019; Sumpter et al., 2016).

Participants held mixed views on the role of staggered closing times in reducing alcohol-related harms in the late night environment. There is a limited evidence base on the impacts of staggered closing times on alcohol-related harms, though a few studies suggest that changes from fixed to staggered closing times are unrelated to changes in violence (Humphreys et al., 2013; Humphreys & Eisner, 2014). Interestingly, participants expressed several views that are not supported by evidence, for example that restricting opening hours in the evening leads to ‘panic drinking’ (i.e. consuming the same amount of alcohol across a shorter-time span) and that ‘vertical’ drinking establishments encourage heavy episodic drinking.

### Areas for further research

Further research is required to investigate how alcohol availability may affect different populations and communities, alongside the role of other outlet characteristics (i.e. longer opening hours, outlet size), and factors including the sale of cheaper alcohol (Horsefield et al., 2023; Morrison et al., 2016). Irresponsible venue operation has been highlighted by qualitative research as an important factor in the limited effectiveness of liquor accords in reducing alcohol-related harms (Curtis et al., 2016). Whilst venue operation was not specifically explored as a mechanism that might influence harms in this study, this would be interesting to explore in future research. Other gaps in the literature include the mechanisms of impact of temporal and spatial availability on population sub-groups including by gender (Fitzgerald et al., 2016; Jackson et al., 2010), different ethnic and cultural groups (Giesbrecht et al., 2016) and socioeconomic status, despite studies showing that on and off-trade outlets cluster in the most deprived areas (Angus et al., 2017; Shortt et al., 2015). Impacts on groups with different types/levels of alcohol consumption are also poorly understood, though there may be a greater association between outlet density and ‘binge drinking’ (Ahern et al., 2016; Arantxa Colchero et al., 2022; Pereira et al., 2013). Recent qualitative research suggests stakeholders hold mixed views on the impacts of limiting late-night trading hours on alcohol consumption ranging from reduced consumption to no change because of the perceived effects of pre-drinking (Miller et al., 2023). This may reflect the limited evidence base exploring the influence of temporal availability on drinking norms and practices, highlighted by a recent qualitative scoping review (Dimova et al., 2023). Addressing these knowledge gaps could improve the translation of research into practice (Ahern et al., 2016), as the lack of evidence has important implications in practice in countries including the UK, where licensing policies and decisions are required to be evidence-based.

### Implications for policy and practice

PHTs and other stakeholders in licensing must articulate evidence-based arguments when making licensing policies and decisions (O’Donnell et al., 2022). Our findings should help them to be clearer about their rationale, and help them to consider how/why specific policies or decisions will have impact. Our findings could be incorporated into guidance and support for PHTs, along with relevant evidence to assist them to construct clearer, more compelling narratives on alcohol availability to assist with local level policy development (such as in developing cumulative impact policies). This could also assist public health stakeholders and advocates to influence national policy, leading to adjustments to licensing laws and guidance (Fitzgerald et al., 2023a). Whilst international research is viewed by some decision makers as irrelevant locally and not, therefore, compelling (Nicholls, 2015), stories drawing on high quality relevant local data and robust mechanisms of impact may hold promise (Fitzgerald et al., 2018b). Finally, our findings can inform theories of change in current and future research evaluating changes in availability (McGill et al., 2015).

### Strengths and limitations

Interviews were in-depth, providing interviewees with ample scope to discuss their views. Our large sample of licensing stakeholders in diverse roles was also a strength of the study. Whilst core questions regarding possible mechanisms of impact were asked across all interviews, our semi-structured, iterative approach meant that not all follow-up questions were explored with all interviewees. Stakeholders found it challenging to articulate with confidence the specific mechanisms by which availability may impact on consumption and harms, which could be interpreted as a study limitation. This likely reflects broader limitations in how prepared PHTs are for a role in licensing and gaps in the international evidence base around links between availability and harm. The findings are limited to mechanisms mentioned by participants in line with the focus of the study on spatial and temporal availability and do not represent an exhaustive list or complete description of possible mechanisms of impact nor of alcohol retailing more broadly. We did not routinely collect data on participant years of relevant experience, so could not examine whether that affected beliefs or mechanisms discussed. Relative to the number of PHT members interviewed, smaller numbers of licensing officers/managers, police staff with a licensing remit, elected members, and licensing lawyers/clerks took part. The findings are necessarily influenced by an England/Scotland perspective, but given the novelty of this work, have potential to contribute to theory, practice, and research internationally.

To conclude, licensing stakeholders, including public health, found it challenging to articulate specific pathways by which changes in alcohol availability may impact on alcohol harms, and were largely unfamiliar with thinking about mechanisms of change. This was most evident in relation to identifying mechanisms by which reduced availability would impact positively on alcohol-related harms. This may be due in part to limitations in the evidence base and may impede their ability to enact or effectively advocate for availability interventions. It may also reflect the time delays involved in achieving change related to reductions in availability. Five overarching mechanisms were identified relating to access/convenience, visibility, premises and area-level norms, affordability, and management of the night-time economy. Current evidence underpinning these mechanisms is mixed and merits further critical scrutiny especially for specific subgroups and contexts. Whilst reducing or preventing increases in availability is a long-term goal, our findings could inform licensing policy, decisions, and advocacy through increased use of the evidence base in decision-making.

## Supplementary Material

Supplemental Material

Supplemental Material

## Appendix 1: Membership of the ExILEnS consortium

Niamh Fitzgerald^1,2^*; Matt Egan^3^; Rachel O’Donnell^1^; James Nicholls^4^; Laura Mahon^1,2,5^; Frank de Vocht^6,7,8^; Cheryl McQuire^6,7^; Colin Angus^2,9;^ Richard Purves^1^; Madeleine Henney^9^; Andrea Mohan^10^; Nason Maani^2,3,11^; Niamh Shortt^2,12^; Linda Bauld^2,13^.

*Principal Investigator

^1^ Institute for Social Marketing & Health, University of Stirling

^2^ SPECTRUM Consortium, UK

^3^ Department of Public Health, Environments and Society, London School of Hygiene & Tropical Medicine

^4^ Faculty of Health Sciences and Sport, University of Stirling

^5^ Alcohol Focus Scotland

^6^ Population Health Sciences, Bristol Medical School, University of Bristol, UK

^7^ NIHR School for Public Health Research, UK

^8^ NIHR Applied Research Collaboration West

^9^ School of Health and Related Research, University of Sheffield

^10^ School of Health Sciences, University of Dundee

^11^ Boston University School of Public Health, USA

^12^ School of GeoSciences, University of Edinburgh

^13^ Usher Institute, University of Edinburgh
