# Supplementary material for: Mechanisms of impact of alcohol availability interventions from the perspective of 63 diverse alcohol licensing stakeholders: a qualitative interview study
Source: Drugs (Abingdon Engl). 2023 May 4;31(3):338–47. doi: 10.1080/09687637.2023.2205991 (PMC11147450; doi:10.1080/09687637.2023.2205991)
Supplement: Supplemental Material [file IDEP_A_2205991_SM1609.docx]

Supplementary Table 2: Summary of online discussion group interview questions on mechanisms of change in relation to alcohol availability interventions

| **Reducing temporal availability** |
| --- |
| **(A) Restricting open hours late at night**  In what ways do you think this policy area could impact on the following harms:   - Impact on consumers (and how consumers might adapt behaviours accordingly) - Impact on harms - Impact on services and costs - Impact on denormalisation - Impact on businesses - Impact on price - Any other possible impacts/effects   *Follow the same discussion structure for:*  **(B) Restricted opening hours in the mornings**  **(C) Staggered opening times**  Does anyone have any additional thoughts/comments? |
| **Reducing spatial availability** |
| **(A) Preventing new premises opening in overprovided areas**  In what ways do you think this policy area could impact on the following harms:   - Impact on consumers (and how consumers might adapt behaviours accordingly) - Impact on harms - Impact on services and costs - Impact on denormalisation - Impact on businesses - Impact on price - Any other possible impacts/effects   *Follow the same discussion structure for*  **(B) Preventing new premises of certain types (off-licensed premises, pubs etc) from opening in particular areas**  **(C) Preventing new premises from opening in locations close to vulnerable groups (ie addiction centres, hospitals) and/or in disadvantaged areas**  **(D) Reducing number of overall outlets from opening (not possible with current UK licensing systems)**  Does anyone have any additional thoughts/comments? |
