# Supplementary material for: Mechanisms of impact of alcohol availability interventions from the perspective of 63 diverse alcohol licensing stakeholders: a qualitative interview study
Source: Drugs (Abingdon Engl). 2023 May 4;31(3):338–47. doi: 10.1080/09687637.2023.2205991 (PMC11147450; doi:10.1080/09687637.2023.2205991)
Supplement: Supplemental Material [file IDEP_A_2205991_SM1608.docx]

Supplementary Table 1: Summary of interview questions on mechanisms of change in relation to alcohol availability interventions

| Licensing Interventions (processes, policies, decisions) |
| --- |
| a) Declining or granting licences  -How does declining/granting licences achieve reductions in harm?  -How does declining/granting licences achieve reductions in alcohol consumption?  b) Opening hours  -How does amending opening hours lead to reduced alcohol harm?  -How would amending opening hours influence people’s behaviour?  c) Cumulative Impact Zones/Overprovision Policies  -How would a cumulative impact zone/overprovision policy reduce harm?  How do you think it influences the nature of premises in the area?  -How does density of outlets lead to reduced harm?  d) Premises types  -What kind of premises are most/least harmful? Why is that?  -Why do you focus on that premise type? Do you think that influences a particular group or behaviour? How? What might the unintended consequences be?  -Do on and off-licences affect harm in different ways? And for different people?  e) Licence conditions  -How do licensing conditions influence behaviour? Whose behaviour do they affect?  -Why do you focus on that licensing condition? Do you think that influences a particular group or behaviour? How does that reduce harm? |
